# Predictors of shoulder dystocia at the time of operative vaginal delivery: a prospective cohort study

**DOI:** 10.1038/s41598-023-29109-7

**Published:** 2023-02-15

**Authors:** Hanane Bouchghoul, Jean-François Hamel, Aurélien Mattuizzi, Guillaume Ducarme, Alizée Froeliger, Hugo Madar, Loïc Sentilhes

**Affiliations:** 1 Department of Obstetrics and Gynecology, Bordeaux University Hospital, Place Amélie Raba Léon, 33076 Bordeaux, France; 2 Clinical Research Center, Angers University Hospital, Angers, France; 3 Department of Obstetrics and Gynecology, General Hospital, La Roche Sur Yon, France

**Keywords:** Neonatology, Reproductive signs and symptoms, Risk factors

## Abstract

Our aim was to identify factors associated with shoulder dystocia following an attempted operative vaginal delivery (aOVD) in a prospective cohort study and to evaluate whether these factors can be used to accurately predict shoulder dystocia by building a score of shoulder dystocia risk. This was a planned secondary analysis of a prospective cohort study of deliveries with aOVD at term from 2008–2013. Cases were defined as women with shoulder dystocia following an aOVD defined as a delivery that requires additional obstetric maneuvers following failure of gentle downward traction on the fetal head to effect delivery of the shoulders. Multivariate logistic regression analyses were performed to determine risk factors for shoulder dystocia. Shoulder dystocia occurred in 57 (2.7%) of the 2118 women included. In the whole cohort, women with shoulder dystocia more often had a history of shoulder dystocia (3.5% vs. 0.2%, p = 0.01), and there was a significant interaction between aOVD and gestational age and the duration of the second stage of labor: women with shoulder dystocia more often had a gestational age > 40 weeks and a second stage of labor longer than 3 h specifically for midpelvic aOVD. In multivariable analysis, a history of shoulder dystocia was the only factor independently associated with shoulder dystocia following aOVD (aOR 27.00, 95% CI 4.10–178.00). The AUC for the receiver operating characteristic curve generated using a multivariate model with term interaction with head station was 0.70 (95% CI 0.62–0.77). The model failed to accurately predict shoulder dystocia.

## Introduction

Shoulder dystocia is a neonatal complication that can be associated with significant perinatal morbidity and mortality, even when managed appropriately^1^. Shoulder dystocia occurs in 0.5 to 1% of spontaneous vaginal deliveries^2^ and in 4 to 9% of operative vaginal deliveries^3–5^.

Many retrospective studies have tried to identify risk factors for shoulder dystocia and have shown that multiple risk factors including a history of shoulder dystocia, maternal diabetes, obesity, increased birth weight, induction of labor, prolonged labor, and operative vaginal delivery (OVD) are associated with shoulder dystocia^5–8^. However, all these risk factors have a low predictive value^9^, except for a history of shoulder dystocia^10^. In particular, 50–75% of cases of shoulder dystocia occur in the absence of any risk factors^2,7,11^. Previous studies have failed to predict the occurrence of shoulder dystocia among women with spontaneous vaginal delivery^12^, and therefore shoulder dystocia is often considered as an unpredictable event^1,2,13^.

Nevertheless, at the second stage of labor when the fetus is engaged in the pelvis and there is an indication to facilitate childbirth, caregivers face the choice between attempting an OVD or performing cesarean section. The risk of shoulder dystocia is increased among women who undergo OVD compared with spontaneous vaginal delivery^3,5,9,14–18^, with an increased risk in the case of vacuum compared with forceps delivery^3,5^. Only one retrospective case–control study assessed risk factors for shoulder dystocia in this population of women with OVD and the authors attempted to provide a predictive model^4^. In this retrospective study including 4,000 women, risk factors associated with shoulder dystocia were parity, diabetes, chorioamnionitis, arrested progress as an indication for OVD, vacuum use, and estimated fetal weight > 4000 g. This predictive model did not accurately predict the occurrence of shoulder dystocia (area under the ROC curve 0.73)^4^. However, this study had limitations: deliveries with the fetal head at midpelvic station were excluded, and estimated fetal weight was extrapolated from birth weight not available at the time of OVD^4^. Identification of the risk factors for shoulder dystocia in this specific high-risk subgroup of women with attempted OVD (aOVD) may help obstetricians to make the appropriate choice between aOVD and cesarean section at full dilation. Thus, robust data related to predictors of shoulder dystocia based on prospective studies are lacking.

Our objective was to identify independent antenatal factors associated with the occurrence of shoulder dystocia at the time of aOVD, and to construct a score to predict shoulder dystocia.

## Materials and methods

This was a planned secondary analysis of a prospective cohort study of 2192 deliveries in women with live singleton term fetuses in vertex presentation who underwent aOVD from December 2008 through October 2013 in a tertiary care university hospital with more than 4000 annual deliveries (Angers, France)^19^. The pre-specified study design was to analyze short-term maternal and neonatal morbidity according to fetal head station (midpelvic, low or outlet aOVD)^19^, to prospectively analyze maternal complications at 6 months according to fetal head station, and specifically in midpelvic or low pelvic aOVD (pelvic floor disorders, sexual dysfunction, postpartum depressive symptoms)^20,21^, and to identify independent antenatal predictors at the time of OVD in cases of morbidity^22^.

We included all women with a live singleton pregnancy in vertex presentation at term (≥ 37 weeks of gestation) who underwent OVD, defined by the placement of at least one blade for forceps or spatula, or a vacuum. Exclusion criteria were multiple gestations, small for gestational age (SGA), defined as less than the 10th centile for gestational age on Hadlock curves^23,24^, a known congenital anomaly, vaginal breech delivery and women with missing data regarding shoulder dystocia.

All women received information about our study. All experiments were performed in accordance with relevant guidelines and regulations. The Research Ethics Committee of the University of Angers, France, approved the study (no. 2008) and confirmed that this study did not require written informed consent from patients.

### Clinical procedures

The decision to perform an OVD, the choice of instrument (forceps, Kiwi OmniCup vacuum, Thierry’s spatulas) and the place of delivery (operating room or not) were left to the obstetrician’s discretion. OVDs were performed by either the attending obstetrician or the obstetric resident, under supervision^22^. All women were offered epidural analgesia. The bladder was emptied by catheter before delivery. OVD classification was based on fetal station, defined according to the ACOG classification^25^. Fetal head station was defined by the level of the leading bony point of the fetal head in centimeters at or below the level of the maternal ischial spines (0 and + 1 = midpelvic; + 2 and + 3 = low;  + 4 and + 5 = outlet).

### Variables

Details of the procedures used to manage labor, as well as maternal characteristics, intrapartum variables, and clinical outcomes identified in the immediate postpartum period were collected prospectively by the midwife or obstetrician and pediatrician responsible for the delivery and the child.

### Outcomes

Cases were defined as women who experienced shoulder dystocia at the time of OVD. According to the current international recommendations, shoulder dystocia was defined as a delivery that requires additional obstetric maneuvers following failure of gentle downward traction on the fetal head to effect delivery of the shoulders (McRoberts maneuver, suprapubic pressure, Woods screw maneuver and extraction of the posterior or anterior arm)^1,2,13^. The diagnosis of shoulder dystocia was made at delivery by the care provider, who could be the obstetrician or the midwife.

Severe maternal morbidity was defined by at least one of the following criteria: third- or fourth-degree perineal tears, perineal hematomas, cervical laceration, extended uterine incision at cesarean delivery, severe postpartum hemorrhage (PPH) > 1500 mL^26^, surgical hemostatic procedure, uterine artery embolization, blood transfusion, infection (endometritis, episiotomy infection, wound infection needing surgery), thromboembolic event (deep vein thrombophlebitis or pulmonary embolism), admission to intensive care unit, and maternal death^19,22,27^. Severe neonatal morbidity was defined by at least one of the following criteria: 5-min Apgar score < 7, umbilical artery pH < 7.00, need for resuscitation or intubation, neonatal trauma, intraventricular hemorrhage > grade 2, admission to the NICU (neonatal intensive care unit) for > 24 h, convulsions, sepsis, and neonatal death^19,22,28^. Neonatal trauma was defined by the existence of at least one of the following: fracture of the clavicle or a long bone, brachial plexus injury, or cephalhematoma; scalp hematoma was defined by a collection of blood in the space and tissue between the skull and skin due to damage to scalp vessels^29^, scalp laceration was defined by bruising or excoriation of the skin^29^. Neonatal sepsis was defined as confirmed clinical infection with positive bacteriological tests^30^.

### Statistical analysis

Continuous data were described by their means ± standard deviations and compared by Mann–Whitney tests, and categorical data were described by percentages and compared by Fisher exact tests. A logistic regression model was used both to assess the relationship between the potential risk factors and shoulder dystocia and to build a score of shoulder dystocia risk.

The potential risk factors considered were determined based on the literature^2^. All factors highlighted in the literature as being associated with shoulder dystocia were included in the model, to be sure not to miss any factor. These covariates were fetal head station at time of OVD (mid vs. low), age (≥ 30 vs. < 30 years), BMI (≥ 25 vs. < 25 kg/m^2^), gestational diabetes mellitus, gestational age at delivery (≥ 40 vs. < 40 weeks), induced labor, duration of the second stage of labor (> 3 vs. ≤ 3 h), active phase of the second stage (> 30 vs. ≤ 30 min), prenatal suspicion of macrosomia, multiparity, epidural analgesia, arrested progress (lack of continuing progress for 30 min, in the active phase in the second stage of labor), vacuum, Caucasian and male fetal gender^11,15,31–35^. For the main analysis, no automatic selection procedure was used to avoid any overfitting problem, and no interaction was considered between the covariates. To verify that the potential absence of effects highlighted with this first model was not linked to a lack of power due to the large number of variables included, a sensitivity analysis was carried out by considering a stepwise procedure for selecting the most important covariates.

A second analysis was performed by also considering the potential interactions between OVD, on the one hand, and each of the other potential covariates, on the other hand. In this model, only significant interactions were retained due to parsimony considerations. The linear combinations obtained with each of these models were used as different scores of shoulder dystocia risk. Their characteristics and accuracy in identifying women at risk of shoulder dystocia were determined using the area under the receiver operating characteristic curve (AUC). An AUC of at least 0.80 was considered to represent an accurate prediction^36,37^.

STATA 14.2 software (StataCorp, College Station, TX) was used for all analyses. P values < 0.05 were considered statistically significant.

## Results

**Figure 1:**
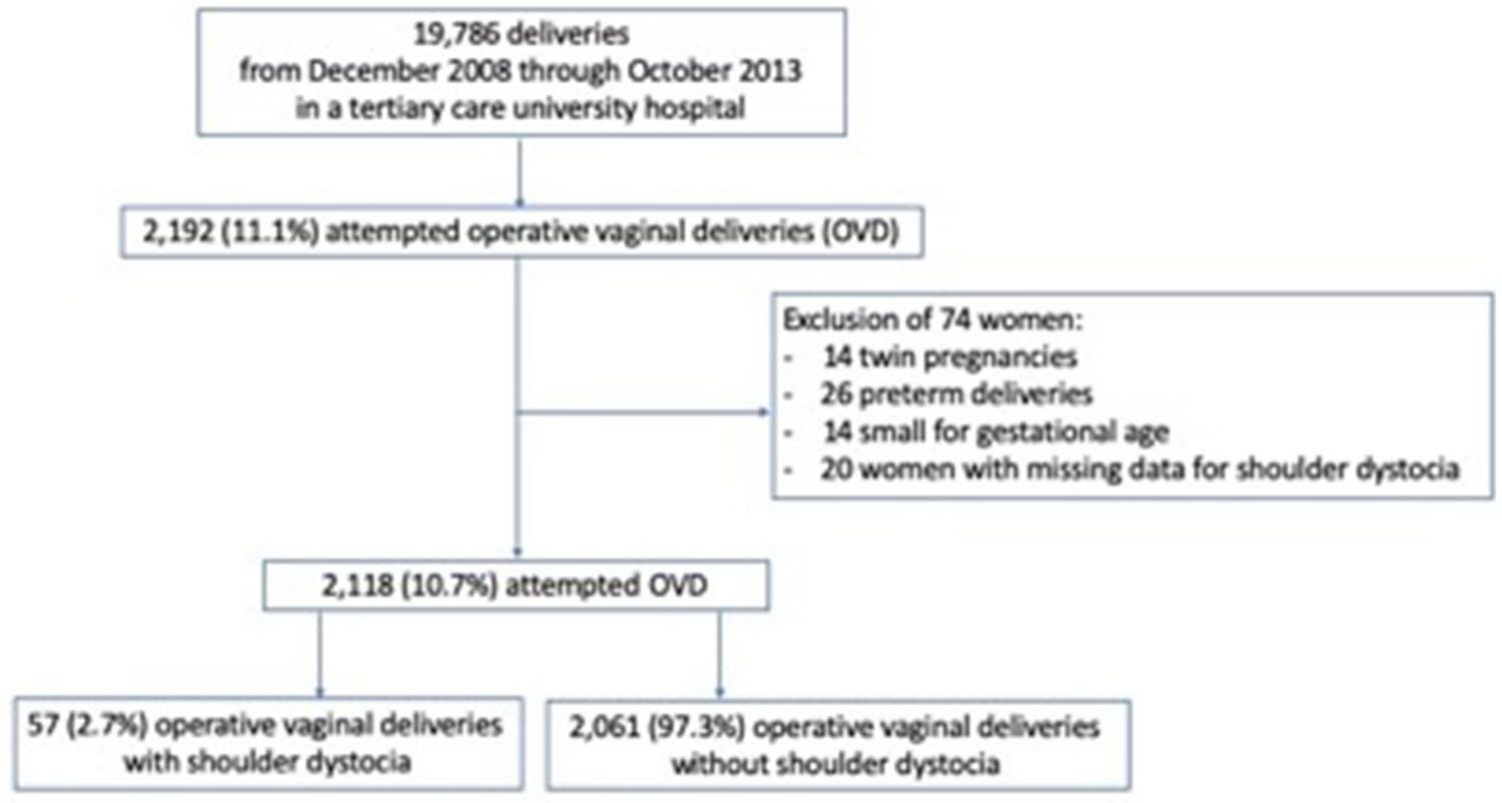
Flow chart.

There were 19,786 deliveries during the study period: 15,836 (80.0%) were vaginal and 3950 (20.0%) were cesarean deliveries. A total of 2192 aOVDs resulted in vaginal delivery in 98.2% of cases (2153/2192) and in cesarean delivery in 1.8% of cases (39/2,192). Within this cohort, 74 women were excluded: 14 twin pregnancies, 26 preterm deliveries and 14 small-for-gestational-age newborns and 20 women with missing data regarding shoulder dystocia (Fig. 1). Therefore, the final sample included 2118 deliveries with an aOVD. Among them, shoulder dystocia occurred in 57 women (2.7%): 23.6% (13/55) after mid-pelvic aOVD, 70.9% (39/55) after low-pelvic aOVD and 5.5% (3/55) after outlet aOVD.

Table 1 details the maternal and labor characteristics and maternal and neonatal outcomes for women who had shoulder dystocia (n = 57) and those who had an OVD without shoulder dystocia after aOVD (n = 2061). Women with shoulder dystocia more often had a previous birth weight above 4000 g (p = 0.003), previous shoulder dystocia (p = 0.01), a gestational age ≥ 40 weeks (p = 0.03) and a birth weight above 4000 g (p = 0.002) (Table 1). Moreover, women with shoulder dystocia had a significantly higher rate of severe neonatal morbidity (26.3% (15/57) versus 10.7% (165/2099); p = 0.001), but not of severe maternal morbidity (8.8% (5/57) versus 7.9% (163/2061); p = 0.80) (Table 2).

**Table 1:** Maternal and labor characteristics and maternal outcomes with and without shoulder dystocia at the time of an attempted operative vaginal delivery.

	Shoulder dystocia (n = 57)	No shoulder dystocia (n = 2061)	p
Maternal and labor characteristics			
Maternal age, years	29.18 + − 5.63	28.13 + − 5.08	0.22
BMI before pregnancy, kg/m^2^	23.50 + − 3.91	22.97 + − 9.85	0.06
Nulliparity	43 (75.44%)	1525 (73.99%)	0.88
Previous cesarean delivery	5 (8.77%)	217 (10.53%)	0.44
Previous birth > 4000 g	4 (7.02%)	20 (0.97%)	0.003
Previous shoulder dystocia	2 (3.51%)	5 (0.24%)	0.01
Gestational diabetes mellitus	4 (7.02%)	155 (7.61%)	1.00
Prenatal suspicion of macrosomia*	6 (10.53%)	159 (7.71%)	0.45
Gestational age at delivery**,** weeks	39.8 + − 1.3	39.4 + − 1.5	0.02
Gestational age at delivery ≥ 40 weeks	38 (66.67%)	1077 (52.26%)	0.03
Induced labor	13 (22.81%)	358 (17.38%)	0.29
2nd stage of labor > 3 h	11 (19.30%)	281 (13.65%)	0.24
Active phase of 2^nd^ stage > 30 min	19 (33.33%)	679 (32.95%)	1.00
Dose of oxytocin (mIU)**	2208.6 ± 2537.9	1692.4 ± 2257.3	0.04
Epidural analgesia	55 (96.5)	1921 (93.3%)	0.58
ACOG classification			
Mid	13/55 (23.6%)	349/2053 (17.0%)	0.34
Low	39/55 (70.9%)	1510/2053 (73.6%)	
Outlet	3/55 (5.5%)	194/2053 (9.4%)	
Indications for OVD			
Non-reassuring FHR only	25 (43.9%)	764 (37.1%)	0.58
Arrested progress only	21 (36.8%)	887 (43.0%)	
Non-reassuring FHR and arrested progress	11 (19.3%)	410 (19.9%)	
Instrument type			
Vacuum	21 (36.84%)	708 (34.40%)	0.78
Forceps	4 (7.02%)	116 (5.64%)	0.56
Spatula	33 (57.89%)	1302 (63.27%)	0.41
Sequential use of two instruments	1 (1.75%)	80 (3.89%)	0.72
Episiotomy	50 (87.72%)	1809 (88.07%)	0.84
3rd- or 4th-degree perineal tears	3 (5.26%)	59 (2.86%)	0.23
Perineal hematomas	0	2 (0.1)	1.00
Blood loss (mL)	481.46 ± 529.36	373.24 ± 370.91	0.13
Postpartum hemorrhage (PPH) (blood loss > 500 mL)	13 (22.81)	328 (15.91%)	0.20
Severe PPH (blood loss > 1500 mL)	2 (3.51%)	35 (1.70%)	0.26
Need for an additional uterotonic agent	2 (8.70%)	42 (3.52%)	0.20
Blood transfusion	2 (3.51%)	36 (1.75%)	0.27
Severe maternal morbidity***	5 (8.77%)	163 (7.91%)	0.80

Continuous data are expressed as means ± standard deviations; discrete data are expressed as n or n (%). Student’s *t* test, χ^2^ test, non-parametric Mann–Whitney test, and Fisher’s exact test were used as appropriate. A *P* value of 0.05 was considered significant.

*Prenatal suspicion of macrosomia: fundal height measurement at delivery > 37 cm and/or ultrasonographic fetal abdominal circumference > 90th p. for gestational age on Hadlock curves.

**Dose of oxytocin: total dose received during labor, including the first and second stages of labor.

***Severe maternal morbidity was defined by at least one of the following criteria: third- or fourth-degree perineal tears, perineal hematomas, cervical laceration, extended uterine incision at cesarean delivery, PPH > 1500 mL^26^, surgical hemostatic procedure, uterine artery embolization, blood transfusion, infection (endometritis, episiotomy infection, wound infection needing surgery), thromboembolic event (deep vein thrombophlebitis or pulmonary embolism), admission to intensive care unit, and maternal death.

**Table 2:** Neonatal outcomes with and without shoulder dystocia at the time of an attempted operative vaginal delivery.

	Shoulder dystocia (n = 57)	No shoulder dystocia (n = 2061)	p
Neonatal outcome			
Birthweight ≥ 4000 g	9 (15.79%)	99 (4.81%)	0.002
5-min Apgar score < 7	1 (1.75%)	20 (0.97%)	0.44
pH < 7.00	1 (1.75%)	31 (1.54%)	0.59
Transfer to NICU	3 (5.26%)	113 (5.50%)	1.0
NICU hospitalization > 24 h	125 (5.9)	3 (7.7)	0.50
Respiratory distress syndrome	5 (8.77%)	75 (3.64%)	0.06
Scalp laceration	5 (8.77%)	110 (5.34%)	0.23
Scalp hematoma	3 (5.26%)	49 (2.38%)	0.16
Pain necessitating drugs	15 (26.32%)	202 (9.83%)	< 0.001
Neonatal trauma*	8 (14.04%)	7 (0.34%)	< 0.001
Fracture of the clavicle	4 (7.02%)	5 (0.24%)	< 0.001
Fracture of a long bone	0	0	–
Brachial plexus injury	4 (7.02%)	2 (0.10%)	< 0.001
Cephalhematoma	1 (1.75%)	7 (0.34%)	0.20
Intraventricular hemorrhage > grade 2	0	0	–
Need for resuscitation or intubation	1 (1.75%)	12 (0.58%)	0.30
Sepsis	1 (1.75%)	18 (0.88%)	0.41
Seizures	0 (0.00%)	5 (0.24%)	1.0
Neonatal death	0	0	–
Severe neonatal morbidity**	15 (26.32%)	221 (10.72%)	0.001

*Neonatal trauma was defined by the existence of at least one of the following criteria: fracture of the clavicle or a long bone, brachial plexus injury and cephalhematoma.

**Severe neonatal morbidity was defined by at least one of the following criteria: 5-min Apgar score < 7, umbilical artery pH < 7.00, need for resuscitation or intubation, neonatal trauma, intraventricular hemorrhage > grade 2, admission to the NICU (neonatal intensive care unit) for > 24 h, convulsions, sepsis, and neonatal death^27^.

**Figure 2:**
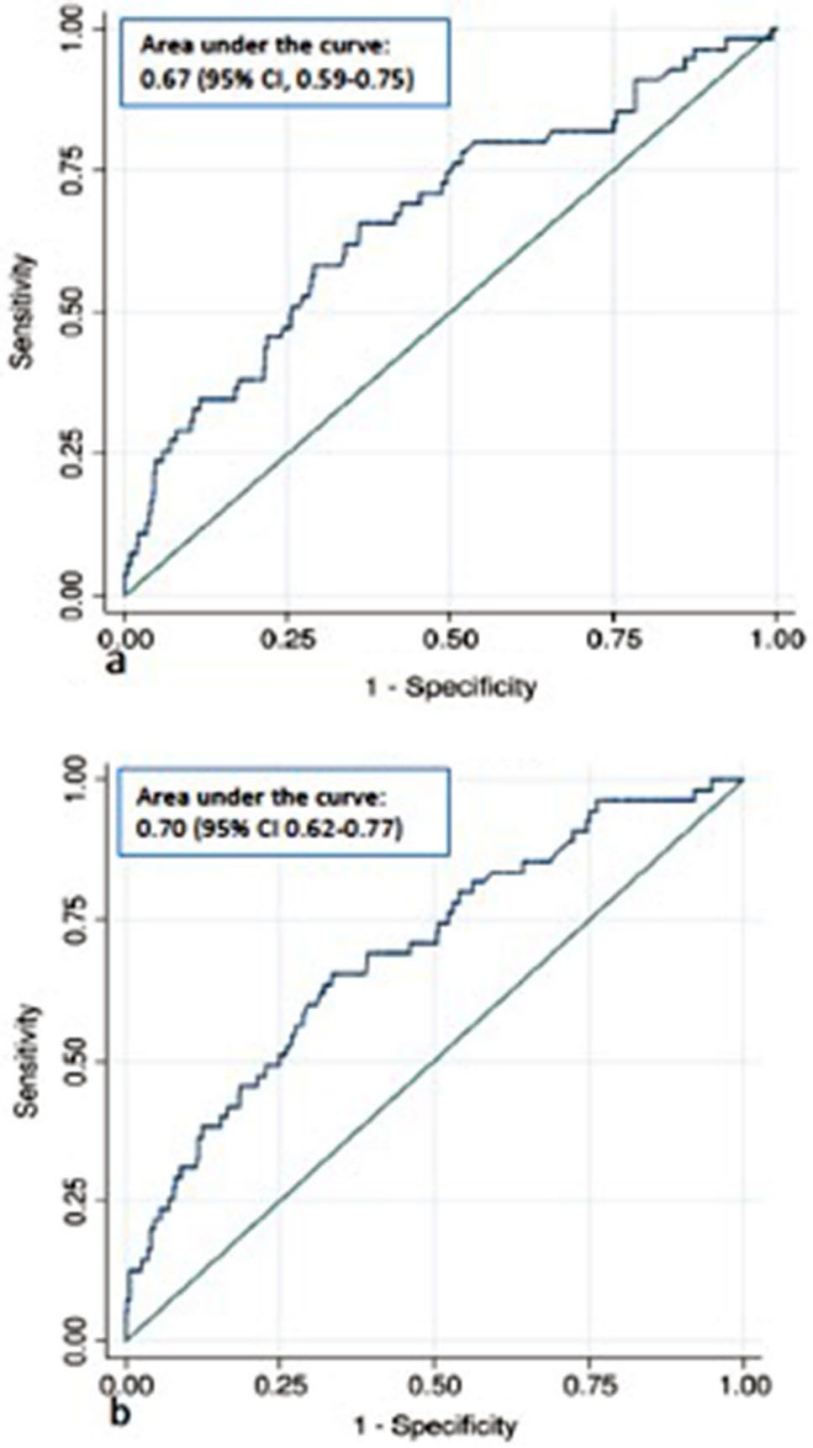
Receiver operating characteristic curve for prediction of shoulder dystocia after attempted operative vaginal delivery according to the prediction model. (**a**) First model without interaction. (**b**) Second model with interaction term with fetal head station.

When considering the main multivariate analysis (i.e. the model without any interactions, and considering the potential risk factors highlighted in the literature), the only independent risk factor was a history of shoulder dystocia (adjusted odds ratio (aOR) 32.88 (95% confidence interval CI 4.88–221.47), p-value < 0.001) (Table 3). This model was characterized by an AUC for the receiver operating characteristic curve of 0.67 (95% CI 0.59–0.75), suggesting poor prediction of shoulder dystocia (Fig. 2a). When the number of variables included in the model was limited by a stepwise procedure to avoid any power issue, the results were quite similar: a history of shoulder dystocia was still the main highlighted risk factor (aOR 22.1, 95% CI 3.9–124.0, p-value < 0.001), the only other highlighted covariate being gestational age at delivery (> 40 weeks of gestation being associated with an aOR of 1.8, 95% CI 1.1–3.3, p-value = 0.04). The AUC of such a reduced model was 0.61 (95% CI 0.53–0.69).

**Table 3:** Factors associated with shoulder dystocia at the time of an attempted operative vaginal delivery (aOVD).

Variables	aOR*	95% confidence interval
First model (without interaction)		
ACOG classification		
Mid compared to outlet aOVD	3.28	0.83–12.96
Low compared to outlet aOVD	1.96	0.57–6.72
History of shoulder dystocia	32.88	4.88–221.47
Age ≥ 30 years	1.55	0.88–2.74
BMI ≥ 25 kg/m^2^	1.34	0.71–2.51
Gestational diabetes mellitus	0.53	0.15–1.86
Gestational age ≥ 40 weeks	1.69	0.94–3.03
Induced labor	1.36	0.71–2.60
2nd stage of labor > 3 h	1.67	081–3.45
Active phase of 2nd stage > 30 min	0.93	0.50–1.72
Prenatal suspicion of macrosomia**	0.98	0.36–2.66
Multiparity	0.72	0.36–1.45
Epidural analgesia	2.08	0.46–9.46
Mother’s height above 160 cm	1.05	0.54–2.05
Instrument type		
Forceps	0.76	0.16–3.68
Spatulas	0.55	0.16–1.98
Vacuum	0.97	0.28–1.46
Second model with interaction		
History of shoulder dystocia	25.78	3.90–170.58
Age ≥ 30 years	1.56	0.88–2.79
BMI ≥ 25 kg/m^2^	1.38	0.73–2.60
Gestational diabetes mellitus	0.59	0.17–2.08
Gestational age ≥ 40 weeks		
If mid aOVD	13.28	1.65–107.02
If low aOVD	1.16	0.61–2.20
Induced labor	1.36	0.70–2.62
2nd stage of labor > 3 h		
If mid aOVD	6.05	1.65–22.21
If low aOVD	1.10	0.44–2.74
Active phase of 2nd stage > 30 min	0.90	0.48–1.69
Prenatal suspicion of macrosomia**	0.99	0.36–2.71
Multiparity	0.72	0.36–1.45
Epidural analgesia	2.36	0.52–10.76
Mother’s height above 160 cm	0.96	0.49–1.89
Instrument type		
Forceps	0.81	0.17–3.89
Spatulas	0.63	0.18–2.19
Vacuum	1.12	0.32–3.93

*All factors highlighted in the literature as being associated with shoulder dystocia were included in the model for adjustment. These covariates were fetal head station at time of OVD (mid vs. low), age (≥ 30 vs. < 30 years), BMI (≥ 25 vs. < 25 kg/m^2^), gestational diabetes mellitus, gestational age at delivery (≥ 40 vs. 40 weeks), induced labor, duration of second stage of labor (> 3 vs. ≤ 3 h), active phase of second stage of labor (> 30 vs. ≤ 30 min), prenatal suspicion of macrosomia, multiparity, epidural analgesia, arrested progress, vacuum, Caucasian and male fetal gender^11,15,30–34^.

**Prenatal suspicion of macrosomia: fundal height measurement at delivery > 37 cm and/or ultrasonographic fetal abdominal circumference > 90th percentile for gestational age on Hadlock curves.

When considering a model with potential interactions between OVD, on the one hand, and each of the other potential covariates, on the other hand, two different interactions could be highlighted: one between OVD and gestational age (p-value = 0.03) and the other between OVD and second stage duration (p-value = 0.03). In addition to the effect of a history of shoulder dystocia, this model highlighted the impact of gestational age > 40 weeks and of a second stage of labor longer than 3 h specifically in the midpelvic OVD population (aOR respectively 13.28 (95% CI 1.65–107.02), and 6.05 (95% CI 1.65–22.21)) (Table 3). In this model, a history of shoulder dystocia remained independently associated with shoulder dystocia following aOVD (aOR 25.78 (95% CI 3.90–170.58)) (Table 3). The AUC for the receiver operating characteristic curve generated using this final regression was 0.70 (95% CI 0.62–0.77), confirming the poor ability of a score to identify women at high risk of shoulder dystocia (Fig. 2b).

## Discussion

Our study has shown in women with an aOVD that the factors independently associated with shoulder dystocia were a history of shoulder dystocia and, in the case of midpelvic aOVD, a second stage of labor longer than 3 h and gestational age ≥ 40 weeks. No valid predictive model of shoulder dystocia at the time of OVD was identified.

A history of shoulder dystocia is a strong and well-known independent risk factor in spontaneous vaginal deliveries with an incidence of recurrent shoulder dystocia ranging between 1.3 and 25%^10,38^ and an odds ratio between 4 and 6^14,34,39^. A history of shoulder dystocia was also the main risk factor of recurrent shoulder dystocia among women with OVD in our study, but interestingly with an incidence of 3.5% and a 25-fold higher risk. However, a history of shoulder dystocia is not an indication of cesarean section for subsequent pregnancies. Regarding the increased risk of recurrence^38^, these patients must be carefully assessed and induction of labor can be proposed at a gestational age earlier than the gestational age at delivery of the index pregnancy.

Moreover, we have shown that gestational age after 40 weeks and a second stage of labor longer than 3 h were associated with shoulder dystocia in the case of midpelvic aOVD. In a large population-based registry study involving 2,014,956 vaginal deliveries in Norway, increased gestational age and prolonged labor (defined as lasting more than 24 h) were also shown to be associated with increased risk of shoulder dystocia independently of other risk factors^34^. In a case–control study including 8010 nulliparous women, the combination of fetal macrosomia, a second stage of labor longer than 2 h, and the use of OVD were associated with shoulder dystocia^17^.

However, in the case of operative deliveries, the association between fetal head station and shoulder dystocia has been little studied. To date, in the only retrospective study that has assessed risk factors for shoulder dystocia among women with operative deliveries, women with midpelvic station instrument application were excluded^4^. Nonetheless, the fetal head at midpelvic station seems to be associated with a higher risk of shoulder dystocia. Thus, our study is the first to take into account this key parameter and to demonstrate that shoulder dystocia is associated with gestational age after 40 weeks and a second stage of labor longer than 3 h only in the case of midpelvic station.

The performance of our prediction models was too weak to be potentially useful for caregivers in daily practice, as has also been the case for other authors. In the study of Palatnik et al. focusing on the subgroup of women with OVD, the prediction model did not accurately predict the occurrence of shoulder dystocia^4^. In fact, in this study, multivariable analysis, parity, diabetes, chorioamnionitis, arrested progress as an indication for OVD, vacuum use, and estimated fetal weight > 4000 g (calculation based on actual birth weight after delivery) remained independently associated with shoulder dystocia. But the AUC for the generated receiver operating characteristic curve was 0.73 (95% confidence interval 0.69–0.77), demonstrating only a modest ability to predict shoulder dystocia before performing an OVD. Other retrospective studies have unsuccessfully tried to build a prediction model in all vaginal deliveries^12,15^. In a retrospective study that aimed to ﻿develop algorithms to calculate the risk of shoulder dystocia among 40,284 consecutive term cephalic singleton pregnancies with spontaneous delivery and OVD^15^, only birth weight, maternal height and OVD were independent antenatal risk factors. This antenatal model had high predictability (area under curve 0.89), but a poor sensitivity of 52.4%. The main limitation of this model was the inclusion in the model of birth weight, a parameter that is unknown at the time of the aOVD. When birth weight was excluded, the prediction of shoulder dystocia was poor.

The first strength of our study was its prospective design in a large well-characterized population of women with aOVD, which allowed us to collect exhaustive and rigorous data, especially concerning all maternal and neonatal characteristics such as estimated fetal weight. The latter key characteristic and antenatal suspicion of macrosomia are rarely available in previous studies, forcing authors to consider birth weight, a postnatal parameter^11,15,31,32,34,35^, or a calculated estimated fetal weight for each woman, but still based on birth weight, as in Palatnik et al.^4^. Another strength of our study is that numerous details were collected regarding all the potential risk factors for shoulder dystocia, including the labor characteristics, which are often unavailable in large retrospective studies, such as fetal head station at the time of an aOVD.

However, our study is not without limitations. Firstly, it reflects the experience of one tertiary university hospital and is generalizable only to other perinatal centers with the same practices (skilled obstetricians, senior obstetrician supervising complex OVD, and daily morning staff meetings). Secondly, as shoulder dystocia is a rare event some of our 95% confidence intervals of the odds ratio are large, thus decreasing the precision of the model provided. Also, given this small number of women with shoulder dystocia, it was not possible to perform a subgroup analysis according to the severity of the shoulder dystocia (i.e. the type and number of maneuvers). In addition, we could not assess whether there were any women who had a planned cesarean section because of a history of shoulder dystocia. Some might consider that our study is limited by the absence of ultrasound assessment of the fetal head station prior to an aOVD, given that several studies have demonstrated that ultrasound examination is more accurate and reproducible than clinical examination in the diagnosis of fetal head position and station^40^. However, as there is no evidence that the use of ultrasound for fetal head station and position improves maternal or neonatal outcome^40^, the majority of authorities do not recommend the routine use of abdominal or perineal ultrasound for assessment of the station, flexion and descent of the fetal head in the second stage of labor^41–45^.

In conclusion, in women with an aOVD, a history of shoulder dystocia and, in the case of midpelvic aOVD, a second stage of labor longer than 3 h and gestational age ≥ 40 weeks are risk factors for shoulder dystocia. However, no valid prediction models of shoulder dystocia were identified.

## Data Availability

The datasets used and/or analysed during the current study are available from the corresponding author on reasonable request.
